# Effect of Layering Technique on Push-Out Bond Strength of Composite Resin to Intracanal Dentin of Primary Anterior Teeth

**Published:** 2018-09

**Authors:** Zohreh Estaki, Hossein Afshar, Sara Ghadimi, Samira Derakhshan

**Affiliations:** 1 Postgraduate Student, Department of Pediatric Dentistry, School of Dentistry, Tehran University of Medical Sciences, Tehran, Iran; 2 Professor, Department of Pediatric Dentistry, School of Dentistry, Tehran University of Medical Sciences, Tehran, Iran; 3 Associate Professor, Laser Research Center of Dentistry, Dental Research Institute, Department of Pediatric Dentistry, School of Dentistry, Tehran University of Medical Sciences, Tehran, Iran; 4 Assistant professor, Department of Oral & Maxillofacial Pathology, School of Dentistry, Tehran University of Medical Sciences, Tehran, Iran

**Keywords:** Composite Resins, Deciduous Teeth, Dentin

## Abstract

**Objectives:**

This in-vitro study aimed to compare the push-out bond strength of composite resin posts packed into the root canal of primary anterior teeth using two different layering techniques.

**Materials and Methods:**

Thirty-two primary anterior teeth were randomly divided into two groups. In group 1, after the preparation of post spaces, a posterior composite resin (Filtek P60) was packed in three horizontal layers by a composite condenser instrument with a cylindrical tip using the horizontal layering technique (HLT). In group 2, this was done using a condenser with a conical tip in three funnel-shaped layers according to the funnel-shaped layering technique (FSLT). Next, the specimens were subjected to push-out bond strength testing. Data were analyzed using t-test and the Kaplan-Meier curves.

**Results:**

The mean±standard deviation (SD) bond strengths of composite resin posts were 8.46±3.45 MPa and 7.7±2.24 MPa for the HLT and FSLT, respectively; the difference was not statistically significant (P=0.46).

**Conclusions:**

The layering technique by which composite resin was packed into the root canal of primary anterior teeth (HLT versus FSLT) had no significant effect on the push-out bond strength of composite resin posts.

## INTRODUCTION

Although dental caries is, for the most part, a preventable disease, it is still the most common chronic disease of childhood [1]. Early childhood caries (ECC) with its aggressive nature, if left untreated, can rapidly involve pulpal tissue leading to dental infection [2]. ECC is known to have psychosocial, physical, and functional impacts. In particular, it affects the children’s nutrition, growth, and development [3].

Anterior esthetic restorations of primary teeth may be categorized as Class III, Class V, Class IV, and full coronal restorations. These restorations can be challenging due to the small size of the teeth, proximity of the dental pulp to the tooth surface, thin enamel, limited surface area for bonding (in severely destroyed teeth), and issues related to the child’s behavior [4].

Full coverage treatment options for primary anterior teeth can be categorized into restorations that are bonded to the tooth and the ones that are fabricated and cemented to the tooth using a luting cement [5]. Bonded restorations include composite resin restorations with or without the use of strip crowns, pedo jacket crowns, and the New Millenium crowns. The other category is made of metal and includes stainless steel crowns (SSC), open-faced SSC, pre-veneered SSC, and Pedo Pearls [5]. Little scientific support exists for any of these techniques, and most of the evidence is regarded as experts’ opinion [4].

In severely carious teeth, endodontic treatment and the use of intracanal posts are inevitable. Intracanal retention in primary anterior teeth can be achieved by several techniques including the use of composite resin posts. The composite resin short-post technique was reported by Kenny et al in 1986 [6]. This simple technique could be designed in the forms of tapered posts (without any undercut) or mushroom posts (by creating an undercut around root canal walls). The latter increases the risk of lateral root perforation and root weakening, especially in young children with thin dentinal root canal walls [6]. Although the Pediatric Restorative Dentistry Reference Manual 2017–2018 by the American Academy of Pediatric Dentistry (AAPD) has not addressed this area, a previous study has stated that composite resin short-post and crown restorations are durable, esthetic, and color stable and can be expected to last until the natural exfoliation of teeth with a normal masticatory function, reasonable diet, and good oral hygiene [6].

Composite resins shrink while polymerizing. An important clinical consideration reducing the effects of polymerization shrinkage is the configuration factor (C-factor). The C-factor is the ratio of bonded surfaces to unbonded surfaces. It is a well-known fact that the higher the C-factor, the greater is the potential for bond disruption due to polymerization effects [7,8]. Internal stresses in preparations with a high C-factor, such as Class I cavities and post spaces, can be reduced by incremental addition of composite resin. Furthermore, the layering technique can influence the C-factor of the cavity and the polymerization shrinkage of the resin composite and subsequently the bond strength [9]. Although some previous studies have evaluated the effect of layering techniques on bond strength to dentin in permanent teeth, this topic has not received sufficient attention in studies on primary teeth. Previous laboratory studies on the push-out bond strength in primary teeth have evaluated the effect of etching time, post space preparations, dentin bonding systems, composite resin type, etc. Therefore, the aim of the present in-vitro study was to evaluate the effect of the layering technique on the push-out bond strength of composite resin posts in primary anterior teeth.

## MATERIALS AND METHODS

The ethics approval for the current study has been obtained from the Ethics Committee of the School of Dentistry of Tehran University of Medical Sciences (TUMS.DENTISTRY.REC.1396.3402). This invitro study was conducted on 32 primary anterior teeth which were extracted due to severe caries within the six months prior to the study.

Written informed consent was obtained from the parents or legal guardians of children whose extracted teeth were used in this study. According to Mosharrafian et al [10] and using two-sample t-test power analysis tab of PASS 11 software (NCSS, LLC, Kaysville, UT, USA), considering α=0.05, β=0.2, mean difference=2.6, and standard deviation (SD)=2.4, the minimum required sample size for each of the two groups was calculated to be 15 samples. Teeth that met the following inclusion criteria were selected:

1. Root resorption by no more than half of the root length.
2. A minimum of 6 mm of root length remained.
3. Absence of internal root resorption in the coronal third of the root canals.

The teeth were immersed in 0.5% chloramine-T solution (Merck KGaA, Darmstadt, Germany) for one week for disinfection and were then transferred to distilled water and stored at 4°C.

### Specimen preparation:

Tooth crowns were cut at the cementoenamel junction (CEJ), perpendicular to the long axis of the teeth using a high-speed handpiece (NSK, Tokyo, Japan) and a fissure diamond bur (No. 138, Jota AG, Switzerland) under water irrigation. The teeth were randomly divided into two groups such that teeth with different root canal diameters were equally distributed in the two groups.The root canals were instrumented to 1 mm short of the working length using three sizes of K-files (Mani Inc., Tokyo, Japan) after the initial file and were irrigated with saline. The root canals were dried with paper points (Gapadent, Tianjin, Korea), and to simulate the clinical conditions, were filled with Metapex paste (Meta Biomed Co. Ltd., Cheongju, South Korea) 1 mm short of the working length and 4 mm apical to the level of cutting.

**Fig. 1:**
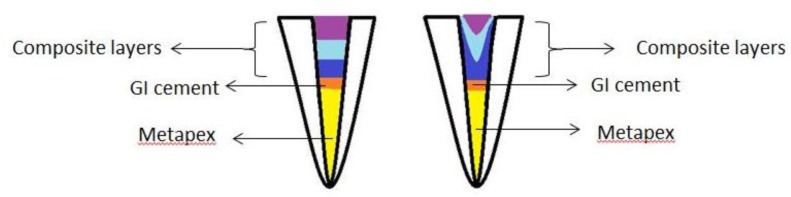
Schematic representation of the HLT (horizontal layering technique; left) and FSLT (funnel-shaped layering technique; right); GI=Glass-Ionomer

A thin layer (approximately 1 mm) of a self-cure glass ionomer cement (GC, Tokyo, Japan) was applied over the Metapex using a condenser in order to obtain a proper apical seat for condensation of composite resin. The 3-mm space available for placement of composite in the root canal was cleaned using a low-speed handpiece (NSK, Tokyo, Japan) and a round carbide bur (C1.RA.016, Jota AG, Switzerland). Next, the following steps were carried out in each group:

In group 1 (the horizontal layering technique, HLT), the root canals were irrigated with normal saline and dried. They were then etched with an acid-etchant (Scotchbond, 3M ESPE, St. Paul, MN, USA) for 7 seconds, rinsed for at least 10 seconds with air/water spray according to the manufacturer’s recommendation, and slightly dried with cotton pellets (for wet bonding). Two layers of Single Bond 2 bonding agent (3M ESPE, St. Paul, MN, USA) were applied, air-dried gently for 3–5 seconds (each layer separately), and light-cured (Guilin Woodpecker Medical Instrument Co. Ltd., Guangxi, China) for 20 seconds. A posterior composite resin (Filtek P60, 3M ESPE, St. Paul, MN, USA) was incrementally applied using a conventional composite condenser instrument with a cylindrical tip in three horizontal layers, and each layer was light-cured for 40 seconds (Fig. 1).

In group 2 (the funnel-shaped layering technique, FSLT), the root canals were treated the same as in group 1. The posterior composite resin (Filtek P60, 3M ESPE, St. Paul, MN, USA) was applied using another type of composite condenser instrument with a conical tip in three increments such that some composite resin ran through the root canal. Each layer was light-cured for 40 seconds (Fig. 1). All the samples were light-cured under similar conditions. The light intensity was 800 mW/cm^2^, and the tip of the curing unit was in close contact with the tooth surface. The light intensity was checked periodically with a radiometer (DigiRate LM-100, Monitex Industrial Co., New Taipei, Taiwan). The samples were then mounted in polyester blocks, and a 1-mm-thick section was made at the middle of the prepared area using a Mecatome (Model T201A; Presi, Paris, France). Photographs were captured by a digital camera (Canon, Eos 600D, Tokyo, Japan) from the two sides of the sectioned specimen while a ruler was placed near each sample, and the root canal periphery was measured using AutoCAD software (2014; Autodesk Inc., San Rafael, USA).

### Push-out bond strength test:

The push-out bond strength test was performed using a universal testing machine (Z050, Zwick/Roell AG, Ulm, Germany). The load was applied to the bonding interface at a crosshead speed of 0.5 mm/minute in an apico-cervical direction using a cylindrical SS plunger. The fracture was recorded in Newton (N) and divided by the cross-sectional area (mm^2^) to calculate the stress at the fracture point and to report the bond strength in Megapascal (MPa). The cross-sectional area was calculated using the formula below [10]:

$$\text{A}=\frac{H\left(A_{1}+A_{2}\right)}{2}$$

Where A_1_ is the circumference of one side of the root canal, A_2_ is the circumference of the other side, and H is the height (mm) of the prepared section of the root.

### Microscopic evaluation and statistical analysis:

The samples were examined under a stereomicroscope (Olympus, Tokyo, Japan) at 40× magnification to determine the mode of failure. The mode of failure was categorized into three groups of adhesive (at the bonding agent-composite interface or at the bonding agent-dentin interface), cohesive (within composite or within dentin), and mixed. The data were recorded, and the push-out bond strength was evaluated using two-sample t-test, log-rank test, and the Kaplan-Meier curves. P≤0.05 was considered statistically significant.

## RESULTS

**Fig. 2:**
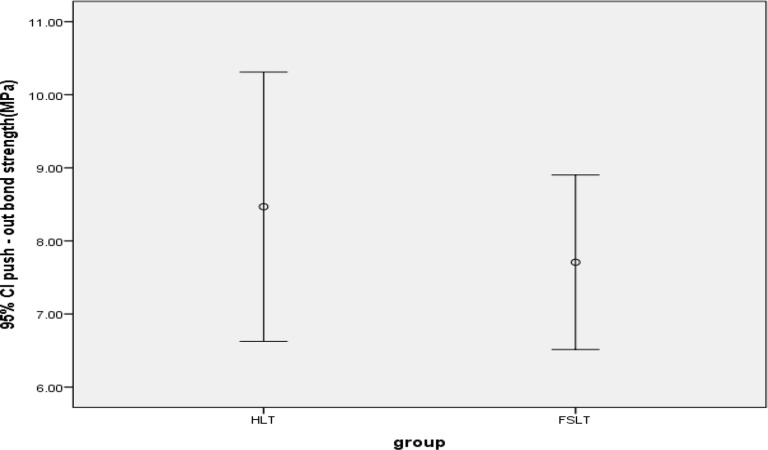
Error bar of the mean and 95% confidence interval (CI) of the push-out bond strength in the two groups.

The mean±SD push-out bond strengths of composite resin posts to intracanal dentin were 8.46±3.45 MPa and 7.7±2.24 MPa for the HLT and FSLT, respectively (Table 1 and Fig. 2). Data analysis using two-sample t-test revealed that there was no statistically significant difference between the two groups (P=0.46).

**Table 1.** The mean bond strength (MPa) and failure modes of the samples in two groups of HLT (horizontal layering technique) and FSLT (funnel-shaped layering technique)

** Group **	** Mean±SD **	** Failure modeN(%) **			

		** Adhesive **	** Cohesive (Composite) **	** Cohesive (Dentin) **	** Mixed **
HLT	8.46±	2	8	0	6
	3.45	(12.5)	(50)		(37.5)
FSLT	7.7±	3	5	0	8
	2.24	(18.7)	(31.3)		(50)

SD=Standard Deviation

**Fig. 3:**
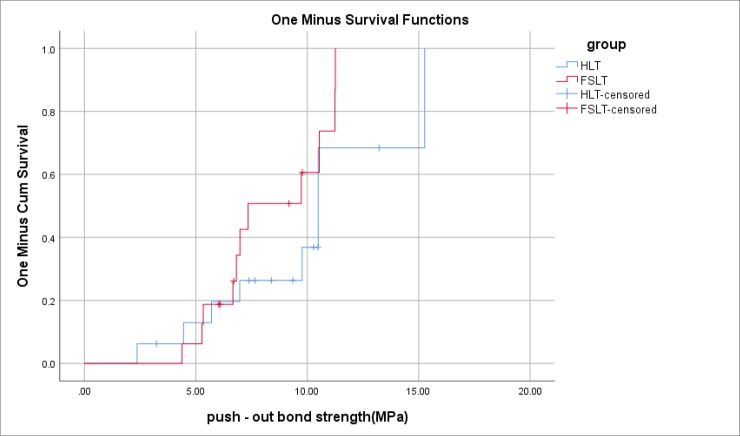
Kaplan-Meier failure functions for the bond strength of the study groups considering cohesive failures as censored data

Analysis of data with log-rank test on the Kaplan-Meier curves with consideration of the failure mode also showed that the mean bond strength was not significantly different between the HLT and FSLT (P=0.192; Table 2 and Fig. 3).

**Table 2.** The mean and median of the push-out bond strength (MPa) in the HLT (horizontal layering technique) and FSLT (funnel-shaped layering technique) using the Kaplan-Meier curves

** Group **	Mean ^*^		** Median **	

	** Estimate **	** Std. Error **	** Estimate **	** Std. Error **
** HLT **	10.454	1.277	10.490	0.393
** FSLT **	8.460	0.681	7.340	2.109
** Total **	9.461	0.719	10.490	0.433

* Estimation is limited to the largest survival time if it is censored

Regarding the failure mode, cohesive (50%) and mixed (50%) fractures were the most frequent fractures in the HLT and FSLT, respectively. Adhesive fractures were the least common failure modes in the two groups. The frequency of the modes of failure in the two groups is shown in Table 1.

## DISCUSSION

The present study assessed the bond strengths of composite resin posts packed into the root canal by two layering techniques, using a push-out model. The push-out test results in a shear stress at the dentin-bonding agent interface as well as the bonding agent-composite resin interface, which is more comparable with stresses under the clinical conditions compared to the linear shear test [11]. Fewer premature specimen failures, minimal laboratory time, and minimized expenses have been associated with the specimens prepared for the push-out test compared to the specimens prepared for microtensile strength testing [12]. In spite of its larger bonded surface and the greater statistical probability of encountering a critical-sized flaw that will lead to failure, the push-out test showed less variability in mechanical testing with a more homogeneous distribution of bond strengths and more reliable data compared to the microtensile strength testing method [12].

Memarpour et al [13] evaluated the retentive strength of composite resin posts, but they did not consider the bonded area and only measured the force required to dislodge the restorations. Since the bonded area directly affects the results of the bond strength test [14], the present study seems to have more accuracy compared to the aforementioned study.

In the current study, the acid-etching duration was considered to be 7 seconds. As stated in previous studies, the reduction of the acid-etching duration of primary dentin by 50% has not only been suggested as a way to maintain adequate bond strengths by the formation of a more homogeneous hybrid layer but also to improve the bond strength in case of single bond application [10,15,16].

Several studies have investigated the push-out bond strength of fiber posts to intracanal dentin of permanent teeth [17–21 ].

The overall assessment of these studies revealed different results according to the type of the posts used, type of luting and bonding systems, and root regions (apical, middle, and coronal thirds) used as specimens. The lower thickness of dentin leading to the proximity of the adhesive to the pulp, the reduced mineral content of dentin, and the greater density and diameters of dentinal tubules in primary teeth, compared to permanent teeth, might contribute to differences in dentin bond strength [15,22]. Furthermore, as peritubular dentin, which is demineralized rapidly during acid treatment, is thicker in primary than in permanent dentin, further decreases in the available bonding substrate might occur [22].

It should be noted that this chemical composition and morphological and structural differences have been exclusively studied and attributed to coronal dentin, and it is difficult, if not impossible, to generalize these findings to intracanal dentin which is the substrate of push-out tests [22]. The inconsistency between the push-out bond strengths of primary and permanent teeth confirms the fact that several additional factors may influence the bond strength.

Based on the results of the present study, this hypothesis that the layering technique of composite resin posts can affect the bond strength was rejected. This may be attributed to the fact that polymerization shrinkage is directly affected by the composite resin volume [23]. It might seem logical to assume that the narrow root canal of primary teeth and the low volume of composite resin applied in each increment can be the cause of this insignificant difference. Contrary to our findings, Nikolaenko et al [7] concluded that for deep Class I cavities, horizontal layering is the most promising way to achieve an acceptable bond to the cavity floor. Different methodological conditions of the two studies (the type of substrate, bonding area, type of composite resin, etc.) may account for these differences. Furthermore, it seems that the FSLT is more technique sensitive compared to the HLT. The greater contact area between the conical tip of the condenser and composite resin in the FSLT during composite resin placement leads to greater likelihood of composite resin sticking to the condenser. This leads to gap formation between root canal wall and composite resin, especially during the application of the first layer. Although the operator put her best effort to carefully adapt composite resin to root canal walls, the microscopic gaps are probably inevitable and consequently affect the results.

Few studies have focused on the push-out bond strength of composite resin posts in primary teeth. Mosharrafian et al [10] concluded that decreasing the etching time from 15 seconds to 7 seconds and preparation of intracanal dentin had no significant effect on the push-out bond strength of composite to intracanal dentin of primary anterior teeth. Values obtained in the cited study are comparable to the results of the corresponding group in our study (the FSLT), indicating relatively similar overall conditions of the two studies.

Afshar et al [24] used a total-etch (Single Bond 2) and two self-etch bonding systems to assess the push-out bond strength of composite resin posts to intracanal dentin of primary anterior teeth and reported that the differences among the three studied groups were not statistically significant. The mean bond strength of the conventional composite in combination with a 5th generation bonding agent was slightly higher than the corresponding value in our study.

Based on the results of another recently published research [25], although bulk-fill composites had lower bond strengths than conventional composites, the difference between them was insignificant. Several factors affecting the bond strength test may account for the variable results of the mentioned studies. These factors include substrate-related variables (source of teeth, type of substrate, depth and location of substrate, direction of enamel rods and dentinal tubules, existence of pulpal pressure, status of the smear layer, storage medium, and time of extraction), specimen-related variables (bonding area and mechanical properties of composites), preparation for bond strength testing (aging protocol as well as thermal and mechanical cycling), and the test method [14].

The analysis of failure modes in the present study revealed that most of the failures were cohesive and mixed, which is in accordance with the results of similar groups in recently published studies [10,24,25]. Baghdadi [26] studied the shear bond strength of a compomer to dentin of primary and permanent molars, using two bonding techniques. It was demonstrated that cohesive failures are not uncommon in primary teeth, and 60% of primary teeth conditioned with phosphoric acid exhibited cohesive failures (30% dentin fracture and 30% composite fracture) [26]. In our study, the bond strength showed no correlation with the failure mode, which is consistent with the findings of de Araujo et al [27]. For example, some samples with low bond strengths showed cohesive failures in the composite resin, while others with higher bond strengths exhibited adhesive failures. However, in the HLT, which showed higher mean bond strengths, more cohesive and fewer adhesive failures were observed compared to the FSLT.

## CONCLUSION

1. Based on the results of the present in-vitro study, the layering technique by which composite resin was packed into the root canal of primary anterior teeth had no effect on the push-out bond strength of composite resin posts.
2. Considering that several factors influence the bond strength of composite resin posts, it may seem logical to use the layering technique that improves the C-factor.
